# The Influence of the Level of Monovision upon Early Outcomes Following the Bilateral Implantation of an Enhanced Monovision Intraocular Lens

**DOI:** 10.3390/vision9020041

**Published:** 2025-05-03

**Authors:** Richard N. McNeely, Stephen Stewart, Niraj Mandal, Salissou Moutari, Allon Barsam, Jonathan E. Moore

**Affiliations:** 1 Cathedral Eye Clinic, Belfast BT1 2LS, UK; richard.mcneely@cathedraleye.com (R.N.M.); stephen.stewart@cathedraleye.com (S.S.); niraj.mandal@cathedraleye.com (N.M.); 2 School of Mathematics and Physics, Queens University Belfast, Belfast BT7 1NN, UK; s.moutari@qub.ac.uk; 3 OCL Vision, London W1G 9TF, UK; allon@oclvision.com; 4 College of Health and Life Sciences, Aston University, Birmingham B4 7ET, UK; 5 Department of Ophthalmology, Tianjin Medical University Eye Hospital, Tianjin 300070, China

**Keywords:** extended depth of focus, monofocal plus IOL, monovision, quality of vision

## Abstract

This article provides an assessment of the impact of different levels of monovision upon early visual outcomes and quality of vision (QoV) following the bilateral implantation of enhanced monovision intraocular lenses (IOLs). Consecutive patients implanted bilaterally with the Rayone EMV (Rayner) were recruited. The dominant eye was targeted for emmetropia, and myopia was targeted in the nondominant eye. Patients were categorized based upon the postoperative refractive outcome in the nondominant eye as follows: Group A: −0.50 to −1.0 D (*n* = 40), Group B: <−1.00 = D (*n* = 46). Uncorrected distance (UDVA), intermediate (UIVA), and near (UNVA) visual acuity, and QoV were compared 3 months postoperatively. Binocular UIVA was 0.05 ± 0.10 and −0.01 ± 0.11logMAR (*p* = 0.03) in the two respective groups, and binocular UNVA was 0.23 ± 0.09 and 0.14 ± 0.09logMAR (*p* < 0.001). Day QoV was 8.77 ± 1.33 and 8.13 ± 1.34 for night QoV in group A, and 8.85 ± 0.99 and 7.85 ± 1.35, respectively, in group B. Group A had a lower spectacle independence rate of 55% compared to 89.1%. This IOL provides a satisfactory range of vision with high QoV satisfaction. A postoperative refractive error of −1.0 D or more in the nondominant eye significantly improves binocular UIVA, UNVA, and spectacle independence, without negatively impacting QoV.

## 1. Introduction

There is an increasing demand for spectacle independence in modern lens-based refractive surgery, and with this, new intraocular lenses (IOLs) are constantly being introduced, which subsequently gives the surgeon an ever-increasing catalogue of lens designs to select from. The aim of modern IOL designs is to provide a range of clear vision from distance to near vision to ultimately provide this spectacle independence, while maintaining good contrast sensitivity and producing minimal visual side effects, and there are various lens designs which claim to provide this. Lens designs range from monofocal IOLs using a monovision approach, diffractive and refractive multifocal IOLs, extended depth of focus (EDoF) IOLs, accommodating IOLs, and various mix and match methodologies [1,2,3]. The latest technology which is increasingly being utilised are enhanced monofocal IOLs. Enhanced monofocal IOLs utilise the asphericity of the IOL to increase depth of focus with low changes in intensity and spot size [4], and it is reported that this methodology can increase the zone of focus by between 1 to 1.5 dioptres (D) [5]. Enhanced monofocal IOLs have been found to provide similar distance vision and dysphoptsias to monofocal IOLs but provide superior intermediate vision [6]. With increasing intermediate vision demands such as computer use, these enhanced monofocal IOLs are becoming increasingly popular. Furthermore, these IOLs can be utilised with a monovision approach, where the nondominant eye is targeted for myopia, to further increase the range of clear vision provided and attempt to provide spectacle independence [7]. This methodology can be a very attractive approach to provide spectacle independence in conjunction with the benefits of fewer visual disturbances and good contrast sensitivity provided by refractive enhanced monofocal IOLs.

A new enhanced monofocal IOL, the Rayone EMV (Rayner Intraocular Lenses Limited, Worthing, UK), has been introduced which is a refractive IOL and is designed to be used in a monovision approach with an emmetropic aim in the dominant eye and an offset aim in the fellow eye. The IOL utilises spherical aberration to elongate depth of focus and for a smoother transition between distance and near vision [4]. To our knowledge, there is a paucity of clinical studies on this new IOL, and therefore this study seeks to outline the associated early postoperative outcomes. This study primarily seeks to provide an understanding of the level of myopia targeted in the nondominant eye and its impact upon spectacle independence and overall satisfaction. This will provide information on the safe level of myopia to target, and the level of monovision required with this IOL to enhance the range of vision sufficiently to provide spectacle independence.

## 2. Materials and Methods

Patients who underwent refractive lens exchange or cataract surgery with a bilateral implantation of enhanced monovision IOLs were included in this study. The nondominant eye was targeted for a myopic postoperative refractive error, and the dominant eye was targeted for emmetropia. This study was a retrospective study and utilised only unidentifiable patient data and each patient gave their informed consent for their anonymised data to be submitted for audit and publication. The exclusion criteria were as follows: ocular inflammation, any other ocular pathology, corneal surgery or disease, a history of glaucoma or retinal detachment, neuro-ophthalmic disease, and macular disease.

Prior to surgery, each patient had a full ophthalmological preoperative assessment. Uncorrected (UDVA) and corrected (CDVA) distance visual acuities were evaluated with logarithmic acuity (logMAR) charts, and Radner reading charts were utilised to assess uncorrected intermediate (UIVA) and near (UNVA) visual acuities. Stereopsis (TNO stereo test), Goldmann tonometry, slit-lamp examination, and dilated funduscopy were completed on each patient. Corneal tomography and pupillometry (MS-39 AS-OCT; CSO, Firenze, Italy), retinal optical coherence tomography (Cirrus 4000 OCT; Carl Zeiss Meditec), and biometry (IOLMaster700; Carl Zeiss Meditec AG, Jena, German) were all completed preoperatively. The Haigis formula was used to calculate IOL power when the axial length was 22 mm or more, and the Hoffer Q formula was utilised when the axial length was less than 22 mm. Ocular dominance was determined using the pointing methodology, which included the following steps: patients were asked to align their finger and a light source 6m in the distance; each eye was then occluded and the eye with the least separation was deemed to be the dominant eye. The outcome of the pointing methodology had to agree with the eye used to sight a camera and/or a rifle, as reported by the patient.

All patients were assessed 3 months after IOL implantation. This examination included manifest refraction, UDVA, CDVA, UIVA, UNVA, stereopsis (TNO stereo test), and contrast sensitivity (Pelli-Robson). Any adverse effect was also noted. To further asses functional reading vision including reading speed and duration, the Salzburg Reading Desk was completed as previously described [8]. To assess subjective outcomes, patients completed a purpose-developed quality of vision (QoV) questionnaire [9] as utilised in previous studies [10]. The questionnaire assessed various visual phenomena and dysphotopsias where the patients report their answers on a Likert scale, and pictures are used to aid understanding. In addition, a linear 0 to 10 scale was used to define each patient’s overall subjective QoV. Patient experience was assessed through a purpose-developed satisfaction questionnaire regarding their distance, intermediate and near vision, and their overall satisfaction [10]. Patients were asked to report two daily activities they perform regularly for distance, intermediate, and near working distances. Patients are provided with a list of distance, intermediate, and near tasks to ensure appropriate tasks are selected for the corresponding distances. If a regularly performed task is not on the provided list, the patient-specific task is recorded. The questionnaire is completed with an optometrist to ensure understanding. For each task, patients respond by stating either that their vision is clear or that they have a slight problem, a moderate problem, a severe problem, or an intolerable problem. Patients also reported how their expectations were fulfilled by the operation by responding either that their expectations were not fulfilled at all, that they were sufficiently fulfilled, fulfilled, or more than fulfilled.

Initial use of this IOL followed a more conservative myopic target approach in the nondominant eye, however, after a period of time using this IOL the myopic target increased. With this increased myopic target and biometry error patients would present with differing levels of postoperative monovision. Therefore, consecutive patients were categorized based upon the postoperative refractive spherical equivalent (SE) outcome in the nondominant eye which allowed for the creation of two separate groups. Patients with a postoperative SE of −0.50 to −1.0 D were categorized into Group A, and those with a postoperative SE of >−1.00 D were categorized into Group B. The maximum level of myopia in the nondominant eye was −1.75 D in Group B. The postoperative assessments were then compared between the two groups.

### 2.1. Intraocular Lens

The Rayone EMV (Rayner Intraocular Lenses Limited, Worthing, UK) is an enhanced monofocal IOL which can be used in a monovision approach, aiming for emmetropia in the dominant eye with a myopic offset in the fellow eye. The IOL provides up to 1.5 D range of focus in the spectacle plane with an emmetropic target as reported by the manufacturers. With a myopic offset of 1.0 D, a depth of focus of 2.25 D is produced. The IOL has a unique positive spherical aberration design that enhances the depth of focus and provides a smoother transition between distance and near eyes, with the periphery of the optic behaving as an aberration neutral IOL [4].

The IOL is a single-piece hydrophilic acrylic with an aspheric anterior surface. It has a 6.00 mm optic diameter and a 12.5 mm overall length. The available powers are +10.0 to +30.0 D in 0.50 D increments. The toric version ranges from +10.0 to +25.0 D in 0.50 D increments and has +0.75D, +1.5 D, +2.25 D, + 3.0 D, and +4.75 D cylinder powers.

### 2.2. Surgical Technique

Two experienced surgeons (JM and NM) completed each surgery. The surgeries were performed under Sub-Tenon anaesthesia, with standard on-axis clear corneal phacoemulsification, and the foldable IOL was inserted through a 2.4 mm incision. An anterior capsulorhexis of 5.5 mm defined by Zeiss Callisto Eye (Carl Zeiss Meditec AG) was created, and the IOL was implanted into the capsular bag. The Callisto eye system was utilised to rotationally align toric IOLs. The nondominant eye was operated on first followed by the second eye one week later.

### 2.3. Statistical Analysis

Preoperative and postoperative parameters were reported in means and standard deviations or percentages, and Excel (Microsoft; Redmond, Washington, DC, USA) and R version 4.3.1 (R Core Team 2021) were utilised for statistical analysis. The median and interquartile range were used to outline the Salzburg Reading Desk findings. An independent *t*-test was used when assessing continuous normal data, and the Mann–Whitney U test was used for assessing nonparametric data. For all statistical analyses, the level of significance was *p* < 0.05.

To have a power of at least 80% a sample size of 36 patients is required to detect a clinically significant difference in QoV of 0.6 between the two postoperative groups, assuming a standard deviation in QoV of 0.9 using a two-tailed *t*-test of difference between means with 80% power and a 5% level of significance.

## 3. Results

This study included 95 consecutive patients. Eight patients were excluded because their postoperative refractive error in the nondominant eye did not fall within the parameters for either Group A or B. One patient was removed from Group B because they required neodymium:YAG capsulotomy at 3 months following the operation. Group A consisted of 40 patients with a mean age of 62 ± 8.53 years, and Group B consisted of 46 patients with a mean age of 58 ± 7.60 years. Table 1 outlines the demographics and preoperative clinical data of the patients.

### 3.1. Refractive Outcomes

**Figure 1:**
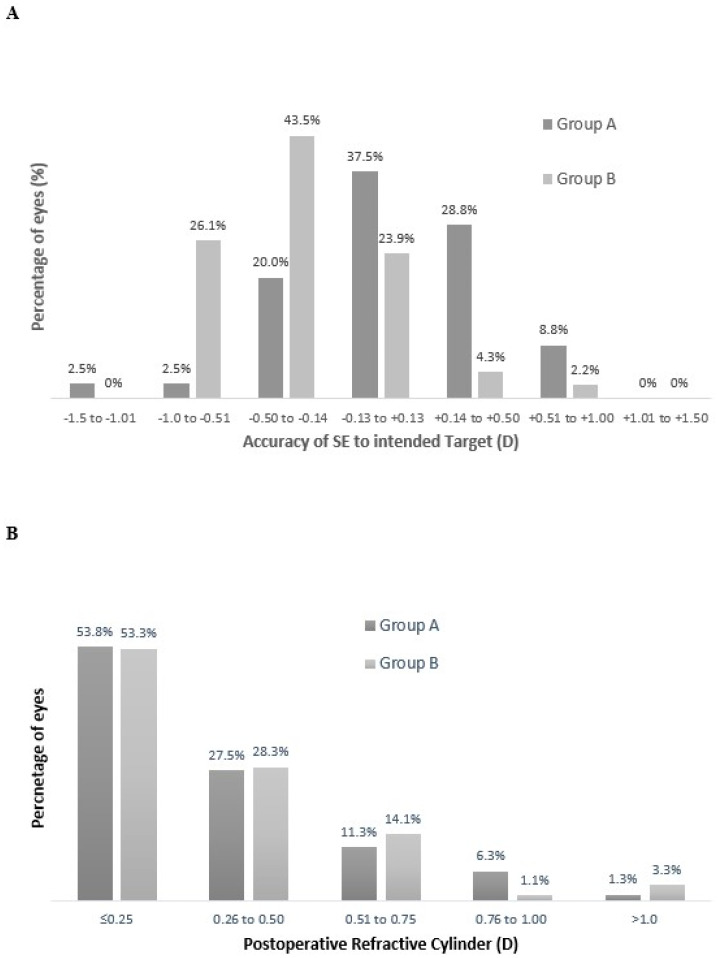
Refractive outcomes of all studied eyes. (**A**) accuracy to the intended spherical equivalent refraction postoperatively for all studied eyes, (**B**) postoperative refractive cylinder (SE = spherical equivalent; D = dioptres).

The refractive predictability analysis of the study group (Group A and B combined) showed that 78% had a refractive accuracy within ±0.50 D of the intended target and 98.8% were within ±1.00 D of the refractive target. Furthermore, Figure 1A outlines that 86.3% of eyes were within ±0.50 D and 97.5% within ±1.00 D of the refractive target in group A. In group B, 71.7% of eyes were within ±0.50 D and 100% within ±1.00 D of the refractive target (Figure 1A). The refractive aim was emmetropia in the dominant eye with 87.5% of dominant eyes within ±0.50 D and 97.5% within ±1.00 D in group A. In group B, 84.8% achieved an SE within ±0.50 D of emmetropia and 100% were within ±1.00 D. Figure 1B displays the postoperative refractive cylinder of the two groups with 81.3% in group A and 81.6% in group B with 0.50 D or less. Table 2 outlines the refractive data for Group A and Group B separately in both the dominant and the nondominant eyes.

### 3.2. Visual Acuity

Table 2 outlines the mean postoperative logMAR values for UDVA, CDVA, UIVA, and UNVA for the two groups. Group B showed significantly better monocular UIVA and UNVA in the nondominant eye, and Group B showed significantly superior binocular UIVA and UNVA. Binocular UDVA was not significantly different between the groups.

**Figure 2:**
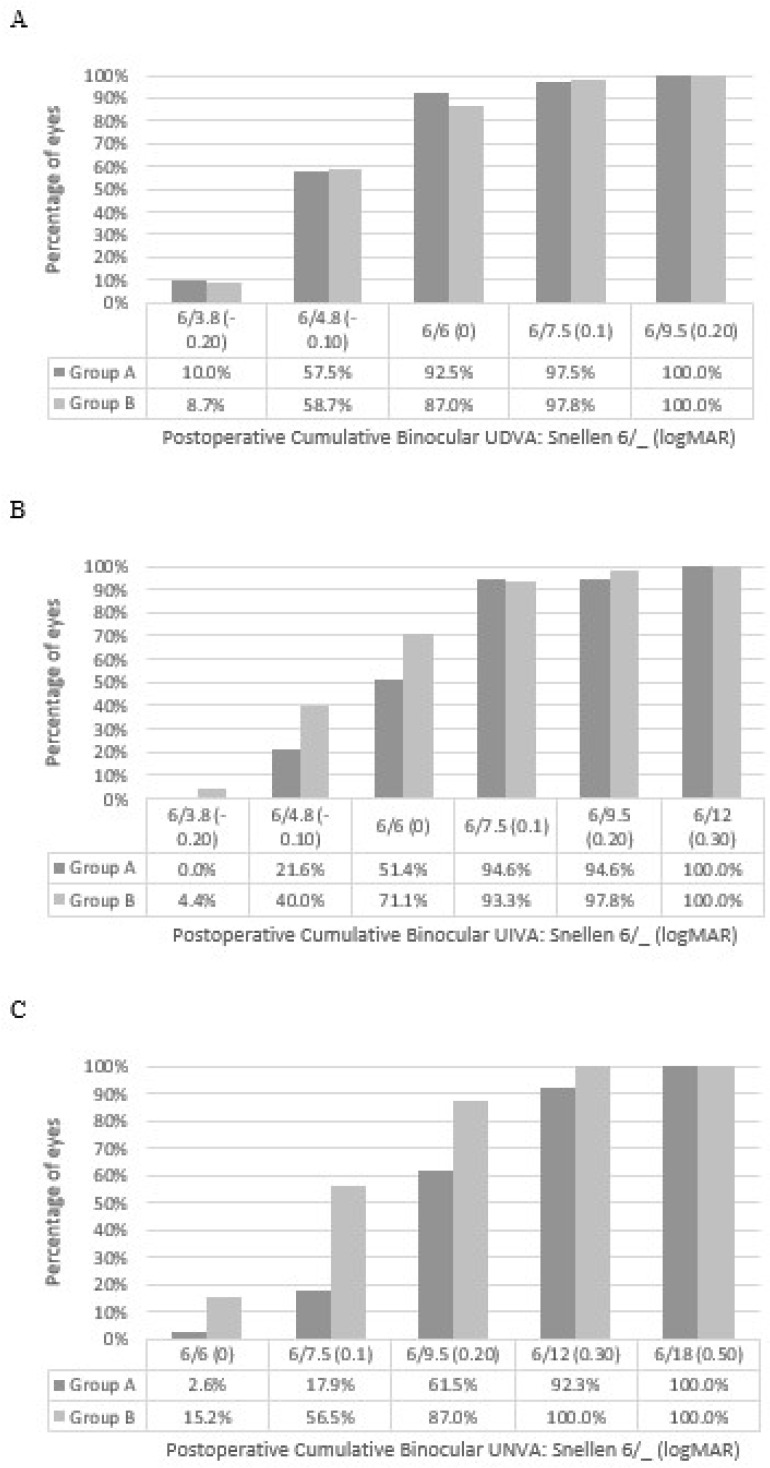
Cumulative binocular uncorrected (**A**) distance, (**B**) intermediate, and (**C**) near visual acuity for group A and B, 3 months postoperatively. (UDVA = uncorrected distance visual acuity).

Figure 2 displays the binocular cumulative UDVA, UIVA, and UNVA.

### 3.3. Reading Performance

Reading performance found with the Salzburg Reading Desk at fixed distances of 40 cm and 66 cm for the two groups is outlined in Table 3 as median values and interquartile ranges. Group B showed better reading acuity, reading speed, and smaller letter size recognition than Group A at 40 cm.

### 3.4. Patient-Reported Outcomes

The QoV questionnaire responses are displayed in Table 4, where there was no statistically significant difference in overall day QoV and night QoV between the two groups. Group B did report more blurred vision symptoms.

The patient satisfaction questionnaire outcomes are highlighted in Table 5. It was found that 55% in Group A and 89.1% in Group B reported complete spectacle independence. In Group A, 77.5% of patients reported to be more than fulfilled or fulfilled with the procedure at 3 months following the operation compared to 78.3% in Group B.

### 3.5. Stereo Acuity and Contrast Sensitivity

There was no significant difference between the two groups with binocular contrast sensitivity with Group A showing 1.64 ± 0.14 compared to 1.70 ± 0.11 in Group B. Furthermore, there was no significant difference in postoperative stereopsis between the two groups.

### 3.6. Complications

One eye required early neodymium:YAG (Nd:YAG) capsulotomy and was therefore removed from the analysis. No other adverse events occurred.

## 4. Discussion

Enhanced monofocal IOLs that manipulate spherical aberration to induce a greater depth of focus are increasingly being utilised in modern refractive IOL surgery. The advantage of these IOLs is that they provide similar distance visual acuity and dysphotopsias compared to monofocal IOLs but provide significantly better intermediate vision [6,11,12]. Furthermore, this approach has been used to minimize spectacle dependence through using a monovision approach. Enhanced monofocal IOLs have been utilised to provide freedom from glasses using a micro-monovision, targeting −0.50 D in the nondominant eye [7,13], or targeting from −0.50 to −1.25 D [14], and appear to provide a range of clear vision with high patient satisfaction.

A new enhanced monofocal IOL, the Rayone EMV, has more recently been introduced. This IOL utilises positive spherical aberration in the centre of the optic to elongate the focus and provide a blend of vision between the two eyes. A myopic offset can be utilised to provide up to 2.25 D of depth of focus [4]. There are limited clinical studies on this IOL, and a particular lack of reporting on subjective patient-reported outcomes, and the level of offset that should be used in the nondominant eye. Therefore, this study sought to outline the early postoperative outcomes with this IOL but in particular to compare the objective and subjective outcomes of patients with a different myopic outcome in the nondominant eye. This will give an understanding of the level of safe monovision to target, determine the level of myopia to target if aiming for spectacle independence, and if a more myopic target negatively impacts overall postoperative QoV.

In this study, patients were then categorized based upon on the 3 months postoperative refractive SE in their nondominant eye. The maximum level of myopia found in the nondominant eye in Group B was −1.75 D, and therefore the SE range of Group B was −1.13 to −1.75 D (Table 2). This allowed for a comparison of both visual and refractive outcomes, and subjective patient responses between two distinct groups.

Figure 1 shows the refractive accuracy to the intended target of all eyes included in this study. It was found that 78% of the study group showed a refractive accuracy within ±0.50 D of the intended target and 98.8% were within ±1.00 D of the refractive target. This is similar to other studies of large cataract outcomes [15] and other presbyopia-correcting IOLs used in refractive surgery [16,17]. This shows a good refractive accuracy to the intended target for this new enhanced monofocal IOL. The refractive aim in the dominant eye of each group was emmetropia and in seven cases the IOL power selected was adjusted based upon the first eye (nondominant) outcome. In group A, 87.5% of eyes were within ±0.50 D of 0 D postoperative refractive SE, as were 84.8% in group B. This represents a high level of accuracy to emmetropia in each group and highlights that the dominant eye was not impacting the near vision outcomes between the two groups. There was no significant difference in postoperative refractive cylinder between the two groups (Table 2), and 81.3% in group A and 81.6% in group B had 0.50 D or less of refractive cylinder (Figure 1B). Each group showed similar and low magnitudes of postoperative refractive cylinder suggesting that neither group was experiencing an increased depth of field due to residual refractive cylinder.

A comparison of the visual outcomes showed that, as expected, the monocular UDVA of the nondominant eyes, where the myopic target is greater, was significantly worse in Group B (Table 2). With the significant difference in UDVA between groups in the myopic eye, it is important to analyse the binocular UDVA, and it was found that both groups had an excellent level of binocular UDVA with no significant difference between them. At 3 months postoperatively, Group A had a binocular UDVA of −0.05 ± 0.09 logMAR and Group B had a binocular UDVA of −0.05 ± 0.08 logMAR (Table 2). This is superior to that found in a recent study of an enhanced monofocal IOL but similar to that of the monofocal IOLs [6]. Figure 2A also shows a similar cumulative percentage of eyes reaching different levels of unaided visual acuity. It appears that the greater amount of myopia in the nondominant eye does not significantly impact the objective binocular UDVA. This is further highlighted in functional UDVA where similar rates of spectacle independence were reported in the two groups. Table 5 shows results of 95% and 100% in the two respective groups for glasses independence for distance vision. The two patients in group A who required glasses for distance vision only did so “occasionally”. This highlights that the greater myopic outcome in the dominant eye does not significantly impact functional distance vision.

A comparison of the monocular UIVA between the two groups showed that, as expected, the monocular UIVA in the nondominant eye was significantly superior in Group B. There was no significant difference between the two groups in the emmetropia eye. A comparison of the binocular UIVA showed that Group B had significantly better binocular UIVA, as shown in Table 2 and Figure 2B. The level of binocular UIVA found in this current study, with both groups, appears to be better than that found in other studies of non-diffractive EDoF IOLs [14], trifocal IOLs [18], and our previous study of a diffractive mix and match IOL approach [19].

When analysing the UNVA between the two groups, similar outcomes were found. Again, the dominant emmetropic eyes had similar UNVA, with superior UNVA in the nondominant eyes of Group B. Likewise, the binocular UNVA was significantly superior in Group B (Table 2), and was superior to other diffractive EDoF IOLs [20], however, it was not as good as the bilateral implantation of trifocal IOLs [21]. Furthermore, Group A also showed superior UNVA compared to non-diffractive EDoF IOLs with a mini-monovision target (−0.50 D) [14]. It was noted that the mean SE in the dominant eye was significantly more myopic in Group B (Table 2), however, this difference did not appear to be clinically significant because there was no difference in monocular UDVA, UIVA, and UNVA between Group A and Group B (Table 2) with the dominant eye.

Additionally, the greater level of myopia in Group B did not show significant differences in stereopsis and contrast sensitivity achieved when compared to group A.

It appears, as expected, that Group B shows significantly better UIVA and UNVA due to the greater myopia in the nondominant eye, but despite significantly more myopia it does not negatively impact objective UDVA, stereopsis, and contrast sensitivity. Both groups displayed excellent binocular UIVA superior to that found in other studies of presbyopia-correcting IOLs. Binocular UNVA in both groups did not appear to reach levels found with bilateral trifocal IOLs. These outcomes seem to support that the use of positive spherical aberration creates a smooth transition between the distance and near eyes and allows for good objective unaided visual acuity without impacting stereopsis and contrast sensitivity.

To understand how the different levels of myopia impact upon subjective postoperative outcomes, patient-reported outcomes were also outlined in this study. Table 4 outlines the outcomes of the QoV questionnaire where no significant difference in overall QoV scores (0, the worst, 10, the best) was found. There was a statistically significant difference in the blurred vision symptom within the QoV questionnaire. The average level of blurred vision in group 2 is low but does appear to be more than group A which is expected due to the higher level of myopia. In group B, no patients reported to be “very” annoyed by blurred vision. The level of blurred vision in Group B does not appear to impact overall satisfaction scores. Additionally, it appears that this new enhanced monofocal IOL does show a lower incidence of dysphotopsias, postoperatively. Direct comparison to diffractive EDoF IOLs in future studies would be beneficial.

The higher degree of myopia in the nondominant eye does not negatively impact the perception of patients’ overall QOV postoperatively.

This study also sought to assess subjective functional vision to determine its relation to objective visual acuity. It was found that Group B showed significantly greater rates of spectacle independence, with 89.1% reporting to be completely spectacle independent, compared to 55% in Group A (Table 5). The level of Group B appears to be comparable to some multifocal IOLs [12,22]. Furthermore, this translated into better functional vision with regular daily tasks performed by patients for both intermediate and near distances (Table 5) in Group B. Group A also appeared to show very good spectacle independence rates compared to other EDoF IOLs [14].

A statistically significant difference in age between the groups in this study was noted (Table 1). An extensive study [23] which compared the objective and subjective outcomes in different age groups following refractive lens exchange and multifocal IOL implantation showed that there was no significant difference in outcomes between the age groups. The average age was 62 in Group A and 58 in group B, and this mean age falls into the two separate groups in the study by Schallhorm et al. [23]. Furthermore, in this current study, the range is similar between the two groups. Further studies with longer-term follow up are required on this IOL to determine the refractive stability of the IOL, the incidence of any further refractive laser surgery to correct residual refractive error, and the rate of posterior capsular opacification requiring neodymium:YAG. Also, direct comparison to other presbyopia-correcting IOLs would be beneficial, in particular to other diffractive and non-diffractive EDoF IOLs.

A limitation of this study is that it does not assess the impact of pupil diameter or corneal spherical aberration upon the postoperative range of vision, and future studies assessing the impact of these parameters are required.

Ocular dominance was assessed using sighting dominance. A study has outlined that ocular dominance is best assessed with tests based upon perceptual preference [24], however, another study showed no clinical difference in using cross monovision [25]. Therefore, sighting dominance assessment was deemed appropriate and to best represent the standard methodology used in many ophthalmology clinical settings due to its simple and quick nature.

## 5. Conclusions

This study found that targeting greater than -1.00 D of myopia is safe and provides excellent levels of spectacle independence without negatively impacting overall QoV, and satisfaction. With increasing intermediate demands, targeting a lower level of myopia still provides excellent distance and intermediate vision and would be a good approach for patients with regular intermediate visual demands, however, they must be selected carefully and counselled regarding spectacle use. In conclusion, the Rayone EMV IOL can be safely used as a monovision approach aiming for spectacle independence, and maintains good UDVA and high overall QoV.

## Figures and Tables

**Table 1:** Demographics and clinical data.

Parameter	Group A	Group B
No. of patients (eyes)	40 (80)	46 (92)
Age (y), mean ± SD (range)	62 ± 8.53 (51 to 79)	58 ± 7.60 (47 to 77)
Gender, male/female (%)	48.8/51.2	26.1/73.9
Axial length (mm), mean ± SD (range)	23.54 ± 1.29 (21.71, 27.23)	23.56 ± 1.52 (20.49, 28.02)
Power of implanted IOL (D), mean ± SD (range)	22.13 ± 3.59 (11.0, 28.0)	21.54 ± 4.53 (10.5, 30)
No. of toric IOLs Power of IOL cylinder (D), mean ± SD (range) Power of IOL cylinder (D), mean ± SD (range)	8 20.19 ± 4.58 (12.5, 25) 1.88 ± 1.13 (0.75, 3.75)	14 20.07 ± 4.53 (11 to 25) 1.88 ± 1.01 (0.75, 3.75)
Clinical, mean ± SD (range)		
Sphere (D)	1.20 ± 2.97 (−8.75 to 6.50)	0.76 ± 3.03 (−10.0 to 5.00)
Cylinder (D)	−0.63 ± 0.70 (−4.50 to 0)	−0.62 ± 0.76 (−4.75 to 0)
MSE (D)	0.69 ± 3.13 (−9.13 to 6.13)	0.44 ± 3.18 (−10.88 to 4.88)
CDVA	−0.01 ± 0.13 (−0.2 to 0.34)	−0.01 ± 0.15 (−0.2 to 0.80)

SD = standard deviation; IOL = intraocular lens; D = diopters; MSE = manifest spherical equivalent; CDVA = corrected distance visual acuity.

**Table 2:** Postoperative clinical data.

Parameter, Mean ± SD (Range)	Postoperative					
	Group A		Group B		*p* Value	
	Dominant	Nondominant Eye	Dominant	Nondominant Eye	Group A to Group B Comparison	
Sphere (D)	0.21 ± 0.39 (−0.50 to +1.50)	−0.61 ± 0.24 (−1.00 to 0)	0.01 ± 0.31 (−0.75 to +1.25)	−1.12 ± 0.22 (−1.50 to −0.50)	0.01	<0.001
Cylinder (D)	−0.38 ± 0.31 (−1.25 to 0)	−0.36 ± 0.31 (−1.00 to 0)	−0.35 ± 0.36 (−1.75 to 0)	−0.43 ± 0.32 (−1.75 to 0)	0.70	0.32
MSE (D)	0.02 ± 0.37 (−1.13 to 1.13)	−0.78 ± 0.18 (−1.00 to −0.50)	−0.17 ± 0.31 (−0.75 to 0.88)	−1.34 ± 0.18 (−1.75 to −1.13)	0.01	<0.001
UDVA (logMAR)	−0.03 ± 0.11 (−0.20 to 0.32)	0.15 ± 0.11 (−0.12 to 0.50)	−0.04 ± 0.10 (−0.20 to 0.20)	0.33 ± 0.14 (0.10 to 0.64)	0.85	<0.001
Binocular UDVA (logMAR)	−0.05 ± 0.09 (−0.20 to 0.20)		−0.05 ± 0.08 (−0.20 to 0.16)		0.87	
UIVA (logMAR)	0.26 ± 0.14 (0 to 0.60)	0.05 ± 0.11 (−0.10 to 0.40)	0.22 ± 0.13 (0 to 0.50)	0 ± 0.11 (−0.20 to 0.30)	0.25	0.02
Binocular UIVA (logMAR)	0.05 ± 0.10 (−0.10 to 0.30)		−0.01 ± 0.11 (−0.20 to 0.30)		0.03	
UNVA (logMAR)	0.47 ± 0.15 (0.20 to 0.80)	0.26 ± 0.10 (0.10 to 0.50)	0.46 ± 0.17 (0.10 to 0.80)	0.16 ± 0.10 (0 to 0.40)	0.77	<0.001
Binocular UNVA (logMAR)	0.23 ± 0.09 (0 to 0.40)		0.14 ± 0.09 (0 to 0.30)		<0.001	
CDVA	−0.08 ± 0.06 (−0.20 to 0.10)	−0.07 ± 0.06 (−0.20 to 0.14)	−0.07 ± 0.07 (−0.20 to 0.20)	−0.07 ± 0.07 (−0.20 to 0.14)	0.44	0.85

SD = standard deviation; D = diopters; MSE = manifest spherical equivalent; UDVA = uncorrected distance visual acuity; UIVA = uncorrected intermediate visual acuity; UNVA = uncorrected near visual acuity; CDVA = corrected distance visual acuity.

**Table 3:** Median binocular near and intermediate distance reading performance with the Salzburg Reading Desk with Group A and Group B.

	Group A		Group B	
	Binocular Near Vision (40 cm)	Binocular Intermediate Vision (66 cm)	Binocular Near Vision (40 cm)	Binocular Intermediate Vision (66 cm)
Reading acuity (logMAR)	0.24 (0.17)	0.03 (0.14)	0.07 (0.12)	0.01 (0.10)
Reading speed (wpm)	93 (34.5)	107 (18)	101.5 (48)	123 (77)
Reading duration (s)	12.1 (4.45)	10.2 (2.05)	10.6 (3.6)	9.6 (4.5)
Letter size	2.00 (0.5)	1.00 (0.23)	1.13 (0.25)	1.00 (0.2)

**Table 4:** Visual Phenomena.

	Group A	Group B	*p* Value
Glare	0.38 ± 0.77 (0, 3)	0.35 ± 0.64 (0, 2)	0.83
Haloes	0.18 ± 0.59 (0, 3)	0.20 ± 0.54 (0, 2)	0.67
Starburst	0.20 ± 0.52 (0, 2)	0.24 ± 0.60 (0, 2)	0.92
Hazy vision	0.18 ± 0.55 (0, 3)	0	0.01
Blurred vision	0.15 ± 0.48 (0, 2)	0.39 ± 0.68 (0, 2)	0.04
Distortion	0.05 ± 0.32 (0, 2)	0	0.29
Double vision	0	0.02 ± 0.15 (0, 1)	0.36
Vision fluctuate	0.13 ± 0.40 (0, 2)	0.13 ± 0.50 (0, 2)	0.62
Depth perception	0	0	1
QoV day	8.77 ± 1.33 (3, 10)	8.85 ± 0.99 (7, 10)	0.76
QoV night	8.13 ± 1.34 (5, 10)	7.85 ± 1.35 (4, 10)	0.34

Visual phenomena calculated on a scale of 0 (not at all) to 3 (very). Values represent mean ± SD (range); QoV is calculated on a scale of 0 (worst) to 10 (best). Values represent mean ± SD (range).

**Table 5:** Postoperative patient-reported outcomes.

Postoperative Assessment		Question				
		How often do you require distance glasses?				
		Never	Occasionally	Quite often	Always	
Group A		95%	5%	0%	0%	
Group B		100%	0%	0%	0%	
		How often do you require reading glasses?				
		Never	Occasionally	Quite often	Always	
Group A		55%	35%	2.5%	7.5%	
Group B		89.1%	10.9%	0%	0%	
		How much difficulty do you have doing a regular task that requires you to see well in the distance?				
		Distance vision is clear	Slight problem	Moderate problem	Severe problem	Intolerable problem
Group A	Activity 1	95%	5%	0%	0%	0%
	Activity 2	85%	2.5%	10%	2.5%	0%
Group B	Activity 1	84.7%	13.0%	2.2%	0%	0%
	Activity 2	73.9%	21.7%	4.3%	0%	0%
		How much difficulty do you have doing a regular task that requires you to see well at intermediate working distances?				
		Intermediate vision is clear	Slight problem	Moderate problem	Severe problem	Intolerable problem
Group A	Activity 1	92.5%	7.5%	0%	0%	0%
	Activity 2	87.5%	12.5%	0%	0%	0%
Group B	Activity 1	95.7%	2.2%	2.2%	0%	0%
	Activity 2	100%	0%	0%	0%	0%
		How much difficulty do you have doing a regular task that requires you to see well at near working distances?				
		Near vision is clear	Slight problem	Moderate problem	Severe problem	Intolerable problem
Group A	Activity 1	67.5%	17.5%	15%	0%	0%
	Activity 2	80.0%	10%	10%	0%	0%
Group B	Activity 1	87%	8.7%	4.3%	0%	0%
	Activity 2	91.3%	8.7%	0%	0%	0%
		How were your expectations fulfilled with the procedure?				
		More than fulfilled	Fulfilled	Sufficiently fulfilled	Not fulfilled at all	
Group A		37.5%	40%	22.5%	0%	
Group B		28.3%	50%	21.7%	0%	

## Data Availability

The datasets used and/or analysed during the current study are available from the corresponding author on reasonable request.

## Abbreviations

The following abbreviations are used in this manuscript:

CDVA	Corrected distance visual acuity
EDoF	Extended depth of focus
IOLs	Intraocular lenses
QoV	Quality of vision
SE	Spherical equivalent
UDVA	Unaided distance visual acuity
UIVA	Unaided intermediate visual acuity
UNVA	Unaided near visual acuity
